# Inferring a District-Based Hierarchical Structure of Social Contacts from Census Data

**DOI:** 10.1371/journal.pone.0118085

**Published:** 2015-02-13

**Authors:** Zhiwen Yu, Jiming Liu, Xianjun Zhu

**Affiliations:** 1 School of Computer Science and Engineering, South China University of Technology, Guangzhou, Guangdong, China; 2 Department of Computing, Hong Kong Baptist University, Hong Kong; University Toulouse 1 Capitole, FRANCE

## Abstract

Researchers have recently paid attention to social contact patterns among individuals due to their useful applications in such areas as epidemic evaluation and control, public health decisions, chronic disease research and social network research. Although some studies have estimated social contact patterns from social networks and surveys, few have considered how to infer the hierarchical structure of social contacts directly from census data. In this paper, we focus on inferring an individual’s social contact patterns from detailed census data, and generate various types of social contact patterns such as hierarchical-district-structure-based, cross-district and age-district-based patterns. We evaluate newly generated contact patterns derived from detailed 2011 Hong Kong census data by incorporating them into a model and simulation of the 2009 Hong Kong H1N1 epidemic. We then compare the newly generated social contact patterns with the mixing patterns that are often used in the literature, and draw the following conclusions. First, the generation of social contact patterns based on a hierarchical district structure allows for simulations at different district levels. Second, the newly generated social contact patterns reflect individuals social contacts. Third, the newly generated social contact patterns improve the accuracy of the SEIR-based epidemic model.

## Introduction

Researchers have recently paid attention to capturing individuals social contacts and social network structures due to their useful applications in the social sciences, ecology, health care, communications, economic sociology [2] [1] [9] [5] [3] [10] and especially epidemic models for infectious disease transmission [17] [16] [15] [4]. Although many studies have examined the relationships among individuals and social network structures, few [20] have considered how to infer the social contact patterns of a hierarchical social structure at the population level directly from census data using a statistical approach.

Every kind of social demographical profile contains a hierarchical structure of social contacts and cross-district contact patterns. such as working, traffic and school patterns, that affect the dynamics of an epidemic. For example, most individuals work in the Central and Western and Wan Chai districts in Hong Kong, but live in the Eastern and Southern districts. If an epidemic were to break out in the Central and Western district, the individuals in the Eastern and Southern districts would be affected immediately. In addition, age- and district-specific patterns have different effects on the spread of disease. Gaining a more complete understanding of epidemic modeling and control requires inferring the hierarchical structure of social contacts from census data.

There are many types of social contacts, such as social media, virtual world and face-to-face contacts. We pay specific attention to face-to-face social contact patterns, as the public health decision department often uses them to capture the dynamic of influenza transmission in the epidemic model. Face-to-face contact suggests physical contact between at least two persons. It includes a mixture of any types of physical contact, such as contact with relatives at home, on public transportation and in the workplace. The approaches applied to identify social contact patterns can be divided into three categories according to different data sources. The first approach involves mining social contact patterns from social networks. For example, Eubank et al. [18] modeled physical contact patterns using the social networks generated by urban traffic simulations built according to actual census, land-use and population-mobility data. Rohani et al. [39] studied the effect of age-structured contact patterns on an epidemic in a detailed contact network. Zelner et al. [47] investigated the effect of social relationships in social networks on the transmission of diarrheal disease. Volz et al. [38] explored the effects of heterogeneous and clustered contact patterns in social networks on the spreading of infectious disease. Lee et al. [30] exploited the structure of social contacts in temporal social networks based on empirical data. Ndeffo Mbah et al. [37] studied the effects of imitation behavior and contact heterogeneity in social contact networks on vaccination coverage. Huerta-Quintanilla et al. [25] modeled social contacts based on social networks in three elementary school case studies. Szell et al. [43] examined the social contact patterns in a large online social network. Son et al. [41] studied a massive online multiplayer social network. Rolls et al. [40] modeled social contacts and designed a contact network for people to explore the spread of hepatitis C. Ventresca et al. [44] constructed a social network based on individuals social contacts as determined by agent-based simulations, and used it to evaluate mitigation strategies. Del Valle et al. [11] proposed a new method of inferring the mixing contact patterns between age groups in social networks. In general, the approaches in the first category can be applied to determine individuals relationships and social contact structures based on their social networks. However, this kind of approach is limited in that it is difficult to construct a social network on a large scale, especially when face-to-face contacts are considered. In addition, the sparse properties of a high dimensional matrix that captures the social contacts from a social network complicates the process of social contact pattern mining.

The approaches in the second category adopt a survey method to collect social contact data from countries such as the United States, France, Belgium, Taiwan, Vietnam and countries in Europe. These data are used to identify social contact patterns. For example, Cauchemez et al. [7] explored the effect of school closure on social contact patterns in relation to influenza transmission based on survey data from France. They also investigated the effect of household contacts on household transmission based on confirmed cases of the 2009 H1N1 virus infection in the United States [8]. Wallinga et al. [45] estimated age-specific transmission parameters using self-reported social contact data. Eames et al. [12] explored the social contact patterns of schoolchildren using survey data from a self-completed questionnaire-based study. Kretzschmar et al. [27] conducted a representative survey involving 7,290 respondents in 8 European countries to capture social contact patterns. They also used social contact patterns to evaluate reproduction numbers based on serological data from five European countries [28]. Glasser et al. [21] considered how to incorporate age-structured contact mixing patterns into the epidemic model using household and cross-sectional serological survey data. Fu et al. [19] investigated contact patterns based on diary-approach surveys conducted in Taiwan. Hens et al. [23] identified social contact patterns based on the data from a two-day population survey conducted in Belgium. Horby et al. [24] studied social contact patterns based on data from a household-based social contact diary in rural Vietnam. Kucharski et al. [29] studied the age patterns of immunity based on mixing data taken from social contact surveys. Melegaro et al. [33] analyzed types of contact based on contact survey data from five European countries. Willem et al. [46] collected data from a social contact survey in Belgium combined with local weather data to investigate the effect of weather conditions and social contact patterns on seasonal influenza transmission during epidemics. Eames et al. [13] conducted a prospective survey to investigate the social mixing patterns of schoolchildren. They also explored social contact patterns according to the results of an Internet-based social contact survey conducted for a cohort of participants over 9,000 times, and examined their simulation effects during the 2009 H1N1v influenza epidemic [14]. Stehle et al. [42] explored the face-to-face contact patterns of children and teachers based on a survey conducted in a French school. Bolton et al. [6] studied the effect of contact definitions on social contact patterns during disease transmission based on survey data from a convenience sample of 65 adults. Mikolajczyk et al. [35] conducted a series of surveys involving university students in Bielefeld from 2003 to 2006 to attain the patterns of direct contact between individuals. These second-category approaches are more suitable for mining social contact patterns on a small scale, such as a community or a small district. They are limited when the survey scale becomes too large, and this increases the human resource and financial costs involved in collecting data.

The approaches in the third category adopt a statistical method to infer social contact patterns directly from the census data published by governments or other authorities. For example, Iozzi et al. [26] identified contact patterns using a synthetic matrix inferred from Italian time-use and routine socio-demographic data. Fumanelli et al. [20] adopted a statistical approach to derive social contact structures from demographic data, which they used to analyze influenza epidemics. In general, the approaches in this category are suitable for mining social contact patterns on a very large scale. In addition, social contact patterns can be easily and correctly inferred from the census data provided by governments and other authorities.

In this paper, we propose a new third-category statistical approach to model a citys population; identify the social contact patterns of the hierarchical social structure at the population level, which are inferred from the detailed census data; and determine hierarchical-district-structure-based, cross-district and age-district-based social contact patterns. We also evaluate the hierarchical structure of social contacts in a simulation of the 2009 Hong Kong H1N1 epidemic to improve the accuracy of the epidemic model. Our work differs from Fumanelli’s [20] in that we provide a more general approach taking into consideration not only the age groups, but also the districts with a hierarchical structure, during the formation of contact matrix.

In summary, the proposed approach has several properties. First, it considers social contacts in a hierarchical district structure. The social contact patterns of districts at different levels form a hierarchical structure that is useful for epidemic control. Second, it considers cross-district contact matrices that account for the contact patterns between individuals in different districts at the same level, which reflects the spatial information during the spread of infectious disease. Third, it considers contact matrices that correspond to both districts and age groups simultaneously. Fourth, it adopts the detailed 2011 Hong Kong census data (released in 2012) to infer the social contact structure. To our knowledge, this paper marks the first time that the detailed 2011 Hong Kong census data has been used to identify social contact patterns.

## Methods

### Census data

To estimate the contact matrices quantitatively, we collected the detailed social census data from Hong Kongs Census and Statistics Department. The most recent census data released by the government was collected in March 2011. The objective of the census is to determine characteristics and trends in the population based on the Hong Kong districts that help the government make decisions. During the 2011 census, 1 in 10 households completed the long questionnaire. Interviews were performed to guarantee the confidence of the provided information. Completed questionnaires were processed and converted into all kinds of statistical tables to ensure the privacy of the respondents information. The statistical tables in the detailed 2011 Hong Kong census data related to our work include the tables for domestic households by district council, household composition and size, domestic households by the sex and age group of the head of the household and by household composition, population by district council constituency area and age, population by sex and district council, age group and economic activity status, persons attending full-time courses by district council, place of study by age group, school type by age, working population by sex, place of work by age group and district council, work type by age and working population by occupation and industry, among others. All of the tables were processed to calculate the contact matrices among different sub-populations.

### Preprocessing of census data

**Fig 1:**
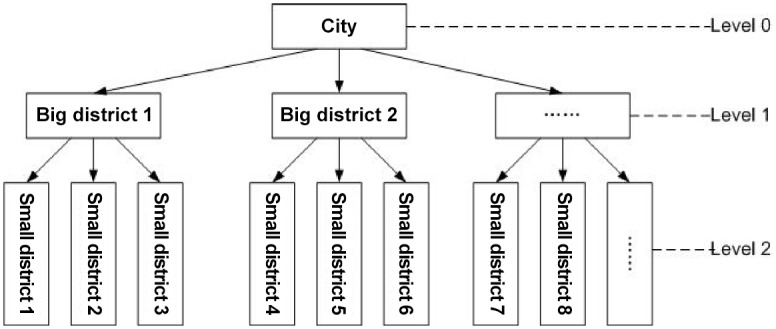
Hierarchical district structure in a city.

**Fig 2:**
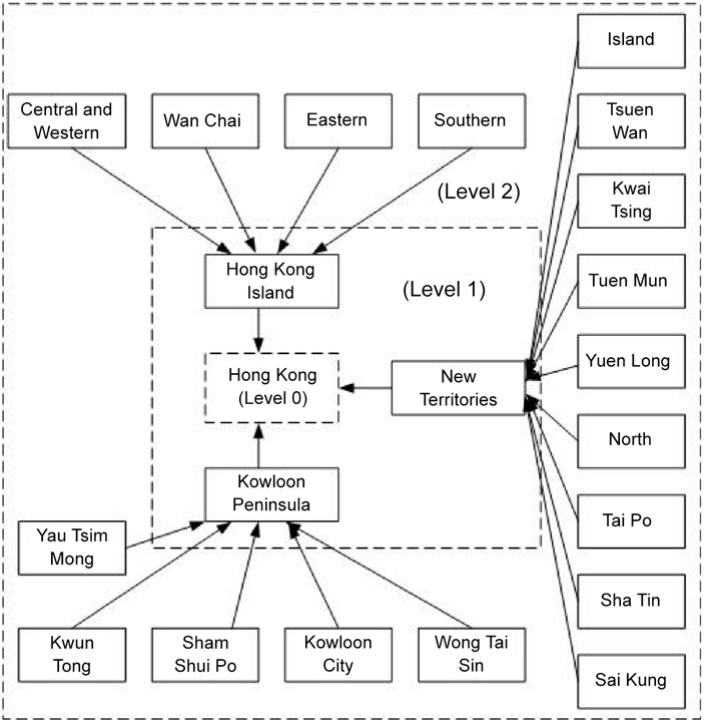
Hierarchical district structure in Hong Kong.

The process of generating synthetic populations in our work is more complicated than that in [20], as we account for the synthetic population based on the hierarchical district structure as shown in Fig. 1, cross-district synthetic population and synthetic population corresponding to both district and age. Fig. 2 illustrates the hierarchical district structure in Hong Kong. Hong Kong (level 0) contains three big districts (level 1), Hong Kong Island, the Kowloon Peninsula and the New Territories, and each of which contains several smaller districts (level 2). Using the detailed social census data, we calculate the number of households in each district, the household compositions, the age of each household member, the activity status of each member, the place of study for persons attending full-time courses, school type in relation to age, working population in relation to place of work, work type in relation to age and working population in relation to occupation. For example, we use the census data related to households by district, household composition and size, households by the sex and age group of the head of the household, and population by district and age to partition individuals into households in different districts. The census data related to working population by district, place of work in relation to age group, persons attending full-time courses by district and places of study in relation to age group can be used to identify individuals who work or study in the same district or across different districts. Statistical data related to population by district, age group and economic activity status allow us to distribute individuals based on activities such as studying, working and staying at home. We use additional census data to generate synthetic populations, especially cross-district synthetic populations.

One of the challenges in the estimation process is how to calculate the cross-district synthetic populations at different levels of the hierarchical district structure. The proposed approach should calculate the average probability of contact between individuals in the *i*-th and *j*-th districts (where *i* ≠ *j*).

**Fig 3:**
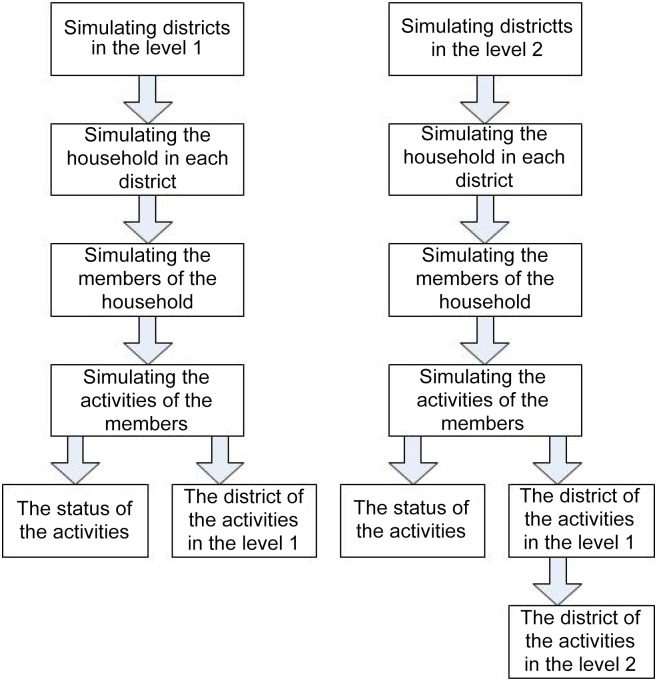
Simulation process in the hierarchical district structure.

Fig. 3 gives an overview of the simulation process for the individuals at different levels of the hierarchical district structure. The proposed approach first estimates the districts, their age structures, the households in each district and the members of each household. It then considers the activities of the household members, including staying at home, staying in the community, studying and working. If the individual stays at home or in the community, the district of the individuals activity is within the same district, which means that the individual belongs to the household or community contact type. If the individual goes to school, the individual and school belong to either the same district or different districts. The probability *p*(*I*) of the individual *I* distributed to each case is calculated as follows:

$$\begin{array}{c} p\left(I\right)=\frac{P_{ij}}{\sum_{j}P_{ij}} \end{array}$$ (1)

where *i*, *j* ∈ {1, …, *r*}, *r* is the number of districts and ∑_*j*_ *P*_*ij*_ denotes the total number of individuals in the *i*-th district who attend school in the *j*-th district. If *i* = *j*, *P*_*ij*_ denotes the number of individuals who attend school in the same district. Otherwise, *P*_*ij*_ denotes the number of individuals who attend school in different districts. Some of the social census data provide only the number of individuals who study in different districts at level 1. Three cases must be considered during the simulation to simulate an individual studying in different districts at level 2. In the first case, the individual and school belong to the same district at level 2. This problem can be solved using the preceding approach. In the second case, the individual and school belong to different districts at level 2, but belong to the same district at level 1. In this case, the proposed approach first determines the level 2 districts that share the same district at level 1, and makes use of the radiation model [22] to distribute the individuals to the corresponding districts at level 2. The traditional radiation model unfortunately does not consider populations consisting of several age groups. The proposed modified radiation model related to age groups is defined as follows:

$$\begin{array}{c} T_{ij}^{h}=T_{i}^{h}\frac{O_{i}^{h}O_{j}^{h}}{\left(O_{i}^{h}+S_{ij}^{h}\right)\left(O_{i}^{h}+O_{j}^{h}+S_{ij}^{h}\right)} \end{array}$$ (2)

where $O_{i}^{h}$ and $O_{j}^{h}$ denote the study opportunities in relation to the *h*-th age group provided by the *i*-th and *j*-th districts, respectively; $S_{ij}^{h}$ denotes the total study opportunities for the *h*-th age group in the circle of radius Γ_*ij*_ centered at the *i*-th district (excluding the source and destination study opportunities); $T_{i}^{h}$ denotes the number of students in relation to the *h*-th age group who study outside the *i*-th district and $T_{ij}^{h}$ denotes the number of students in the *h*-th age group who live in the *i*-th district but study in the *j*-th district, as predicted by the radiation model. In the third case, the individual and school belong to different districts at levels 1 and 2 simultaneously. The proposed approach first assigns the individual to the *i*-th district at level 1 as shown on the right side of Fig. 3, and then distributes the individual to the district at level 2 based on the radiation model [22].

If an individual is working, the proposed approach simulates two scenarios in which the individuals workplace is in the same district and a different district, respectively. The simulation process is similar to that used for individuals studying at school. In summary, the preceding simulation process captures the hierarchical-district-structure-based and cross-district contact patterns.

### Calculation of contact matrix

Two important factors are closely related to the social structure of the population: district and age. District reflects the spatial properties of the population, and age captures its temporal properties. For example, workers in districts with more business centers tend to spend more time with their business partners at their companies, and teenagers prefer to spend more time with their classmates at school. This means that social contacts differ by district and age group. To capture the social and demographic structure of the population, heterogeneous contact patterns among individuals should be identified corresponding to district and age groups. We adopt contact matrices *W* to represent heterogeneous contact patterns, the entries *w*_(*i*−1)*a+*h*, (*j*−1)*a+*k*_ (rewritten as $w_{hk}^{ij}$) of which denote the average contact probability between an individual belonging to the *h*-th age group in the *i*-th district and an individual belonging to the *k*-th age group in the *j*-th district (where *h*, *k* ∈ {1, …, *a*}, *a* is the number of age groups, *i*, *j* ∈ {1, …, *r*} and *r* is the number of districts). Two individuals may share contact if they share the same physical environment [20], such as the household, school, workplace or community. We adopt the same assumption as [20], which states that the mixing in the finest unit, such as the single household or school, is homogeneous. Individuals have four types of social contact patterns: those that occur in the household, school, workplace and community. As a result, district and age group contact matrices are calculated based on four types of contact matrices *W* ^*e*^ (*e* ∈ {1, 2, 3, 4}) related to the household *W* ^1^, school *W* ^2^, workplace *W* ^3^ and community *W* ^4^, respectively. It is reasonable to assume that social contacts in the household and community occur within the same district, and that those related to the school and workplace occur within the same district or across districts. Instead of considering the workplace, we divide working individuals by their occupation and industry, and view those individuals who are in the same occupation and industry as a homogeneous unit.

The proposed approach first calculates the contact probability ${\left(U_{hk}^{ij}\right)}^{edb}$ per individual belonging to the *h*-th age group in the *i*-th district and the *k*-th age group in the *j*-th district (where the superscript (⋅)^*edb*^ denotes the social contacts appearing in the *b*-th unit in the *d*-th district in relation to *e*-th contact type; *b* ∈ {1, …, *B*}; *B* is the number of total units; *d* ∈ {1, …, *r*}; *r* is the number of districts in the corresponding level and *e* is the contact type) as follows:

$$\begin{array}{c} {\left(U_{hk}^{ij}\right)}^{edb}=\left\{\begin{array}{cc} \frac{{\left(P_{k}^{j}\right)}^{edb}-\delta_{hk}^{ij}}{P^{edb}-1} & \;\mathrm{if}\;P^{\mathrm{edb}}>1,\\ 0 & \;\mathrm{if}\;\mathrm{otherwise}, \end{array}\right. \end{array}$$ (3)

$$\begin{array}{c} P^{edb}=\sum_{i=1}^{r}\sum_{h=1}^{a}{\left(P_{h}^{i}\right)}^{edb} \end{array}$$ (4)

where ${\left(P_{h}^{i}\right)}^{edb}$ and ${\left(P_{k}^{j}\right)}^{edb}$ denote the total number of individuals belonging to the *h*-th age group in the *i*-th district and the *k*-th age group in the *j*-th district, respectively. *a* is the number of age groups. $\delta_{hk}^{ij}$ is a Kronecker delta formula and is defined as follows:

$$\begin{array}{c} \delta_{hk}^{ij}=\left\{\begin{array}{cc} 1 & \mathrm{if}\;i=j\wedge h=k,\\ 0 & \mathrm{if}\;\mathrm{otherwise}, \end{array}\right. \end{array}$$ (5)

This means that when the number of individuals is greater than 1, the preceding formula works. Otherwise, ${\left(U_{hk}^{ij}\right)}^{edb}=0$.

The total contact frequency matrix *C* ^*ed*^ in the *d*-th district with the *e*-th contact type is calculated, the entries ${\left(c_{hk}^{ij}\right)}^{ed}$ of which are as follows:

$$\begin{array}{c} {\left(c_{hk}^{ij}\right)}^{ed}=\sum_{b=1}^{B}{\left(U_{hk}^{ij}\right)}^{edb}{\left(P_{h}^{i}\right)}^{edb} \end{array}$$ (6)

We then calculate the average contact frequency matrix *F* ^*ed*^ in the *d*-th district with the *e*-th contact type. Its entries ${\left(f_{hk}^{ij}\right)}^{ed}$ are as follows:

$$\begin{array}{c} {\left(f_{hk}^{ij}\right)}^{ed}=\frac{{\left(c_{hk}^{ij}\right)}^{ed}}{{\left(N_{h}^{i}\right)}^{ed}{\left(N_{k}^{j}\right)}^{ed}} \end{array}$$ (7)

$$\begin{array}{c} {\left(N_{h}^{i}\right)}^{ed}=\sum_{b=1}^{B}{\left(P_{h}^{i}\right)}^{edb}\;\;\;\;if\;\;P^{edb}>1 \end{array}$$ (8)

$$\begin{array}{c} {\left(N_{k}^{j}\right)}^{ed}=\sum_{b=1}^{B}{\left(P_{k}^{j}\right)}^{edb}\;\;\;\;if\;\;P^{edb}>1 \end{array}$$ (9)

where ${\left(N_{h}^{i}\right)}^{ed}$ denotes the total number of individuals belonging to the *h*-th age group in the *i*-th district whose effective social contacts occur in the *d*-th district. ${\left(N_{k}^{j}\right)}^{ed}$ can be defined in a similar way.

We calculate the contact matrix *W* ^*ed*^ in the *d*-th district with the *e*-th contact type, and its entries ${\left(w_{hk}^{ij}\right)}^{ed}$ are as follows:

$$\begin{array}{c} {\left(w_{hk}^{ij}\right)}^{ed}={\left(\phi_{hk}^{ij}\right)}^{ed}{\left(f_{hk}^{ij}\right)}^{ed} \end{array}$$ (10)

$$\begin{array}{c} {\left(\phi_{hk}^{ij}\right)}^{ed}=\frac{{\left(N_{h}^{i}\right)}^{ed}{\left(N_{k}^{j}\right)}^{ed}}{P_{h}^{d}P_{k}^{d}} \end{array}$$ (11)

where $P_{h}^{d}$ is the total number of individuals belonging to the *h*-th age group who live in the *d*-th district and who participate in activities in the *d*-th district, which can be derived from the preceding simulation process. $P_{k}^{d}$ can be defined in a similar way.

If we consider only one district, the contact matrix *W* ^*d*^ in the *d*-th district is calculated. Its entries ${\left(w_{hk}^{ij}\right)}^{d}$ are as follows:

$$\begin{array}{c} {\left(w_{hk}^{ij}\right)}^{d}=\sum_{e}\frac{\omega_{e}{\left(w_{hk}^{ij}\right)}^{ed}}{\sum_{e}{\left(w_{hk}^{ij}\right)}^{ed}} \end{array}$$ (12)

where the values of the parameters *ω*_*e*_ are similar to [20].

If we account for all of the districts at the same level, the contact matrix *W* ^*e*^ with the *e*-th contact type is calculated, and its matrices ${\left(w_{hk}^{ij}\right)}^{e}$ are determined as follows:

$$\begin{array}{c} {\left(w_{hk}^{ij}\right)}^{e}=\sum_{d=1}^{r}\zeta_{hk}^{d}{\left(w_{hk}^{ij}\right)}^{ed} \end{array}$$ (13)

$$\begin{array}{c} \zeta_{hk}^{d}=\frac{P_{h}^{d}P_{k}^{d}}{P_{h}P_{k}} \end{array}$$ (14)

where *P*_*h*_ denotes the total number of individuals in the *h*-th age group and *P*_*k*_ denotes the total number of individuals in the *k*-th age group.

Finally, we calculate the contact matrix *W* in relation to all of the contact types. Its entries $w_{hk}^{ij}$ are as follows:

$$\begin{array}{c} w_{hk}^{ij}=\sum_{e}\frac{\omega_{e}{\left(w_{hk}^{ij}\right)}^{e}}{\sum_{e}{\left(w_{hk}^{ij}\right)}^{e}} \end{array}$$ (15)

In summary, we use the proposed approach to calculate the average contact probability matrix *W* in terms of all of the contact types level by level in the hierarchical district structure, and form a set of hierarchical-district-structure-based contact matrices.

### Contact matrix permutation

To determine the patterns in the contact matrix, we define the permutation operator *π* to re-sort the rows and columns. We consider the first *l* (where *l* is a parameter specified by the user) at the largest contact probability for each row or column, and define an objective function *ζ*_*i*_ for the *i*-th row or column, which is the sum of the first *l* for the *i*-th row or column. We then use the permutation operator *π* to re-sort the rows and columns in the contact matrix in descending order according to the values of the objective function *ζ* as follows:

$$\begin{array}{c} \left(\begin{array}{c} 1\;\;\;,\;2\;\;,\;3\;\;,\ldots ,\;n\\ \pi (1),\pi (2),\pi (3),\ldots ,\pi (n) \end{array}\right) \end{array}$$ (16)

The lines and columns may be rearranged because they are all independent, as all of the districts are independent. This means that there are no relationships between any two neighboring lines or columns. As a result, the rearrangement process based on the contact probabilities provides a chance to capture the patterns among the lines and columns.

### Contact matrix validation

To evaluate the effectiveness of the newly generated contact matrix, we incorporate the contact matrix into the SEIR model [34], which is formulated as follows:

$$\begin{array}{c} \frac{dS_{h}}{dt}=-\chi_{h}\cdot S_{h}\;\;\;\;\;\;\;\;\;\;\;\;\;\; \end{array}$$ (17)

$$\begin{array}{c} \frac{dE_{h}}{dt}=-\varphi_{h}\cdot E_{h}+\chi_{h}\cdot S_{h} \end{array}$$ (18)

$$\begin{array}{c} \frac{dI_{h}}{dt}=-\psi_{h}\cdot I_{h}+\varphi_{h}\cdot E_{h}\; \end{array}$$ (19)

$$\begin{array}{c} \frac{dR_{h}}{dt}=\psi_{h}\cdot I_{h}\;\;\;\;\;\;\;\;\;\;\;\;\;\;\;\; \end{array}$$ (20)

where *S*_*h*_, *E*_*h*_, *I*_*h*_ and *R*_*h*_ denote the compartments of the SEIR model in the age-specified structure, including the susceptible, exposed, infectious and recovered compartments, respectively. *h* ∈ {1, …, *a*} denotes the *h*-th age group. *a* is the number of age groups. *ϕ*_*h*_ is the incubation rate. *ψ*_*h*_ is the recovery rate. *χ*_*h*_ is the infection risk, which is defined as follows:

$$\begin{array}{c} \chi_{h}=\frac{1}{a}\cdot \left(\sum_{k=1}^{a}w_{hk}\cdot \frac{I_{k}}{P_{k}}\right)\cdot \frac{S_{h}}{P_{h}}\cdot \beta_{h} \end{array}$$ (21)

where *w*_*hk*_ denotes the value of the entry in the contact matrix *W* in relation to all of the contact types, and *β*_*h*_ denotes the infection risk. The infection risk *χ*_*h*_ consists of two parts: the risk of infectious contacts determined by *w*_*hk*_ and the generic infection vulnerability *β*_*h*_.

## Results

### Simulation with the detailed 2011 Hong Kong census data

We evaluate the simulation process of the proposed approach using the detailed 2011 Hong Kong census data.

**Fig 4:**
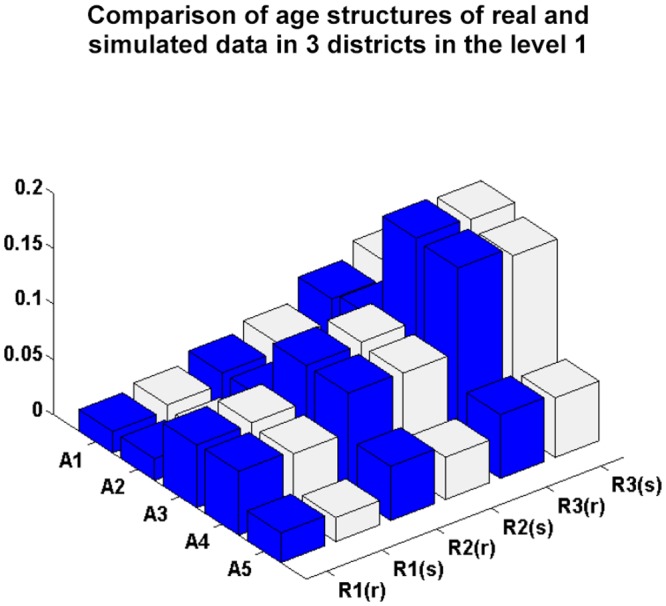
Comparison of the populations of simulated and real data in the districts at level 1, including Hong Kong Island (R1), Kowloon Peninsula (R2), and the New Territories (R3) (where R1(r), R2(r) and R3(r) denote the real data in the three districts, respectively, and R1(s), R2(s) and R3(s) denote the simulated data in the three districts, respectively).

Fig. 4 compares the real and simulated population data in relation to the age structures in the different level 1 districts. The numbers of simulated individuals in each age group in each district are close to the number of real individuals, indicating that the simulation process is successful.

**Fig 5:**
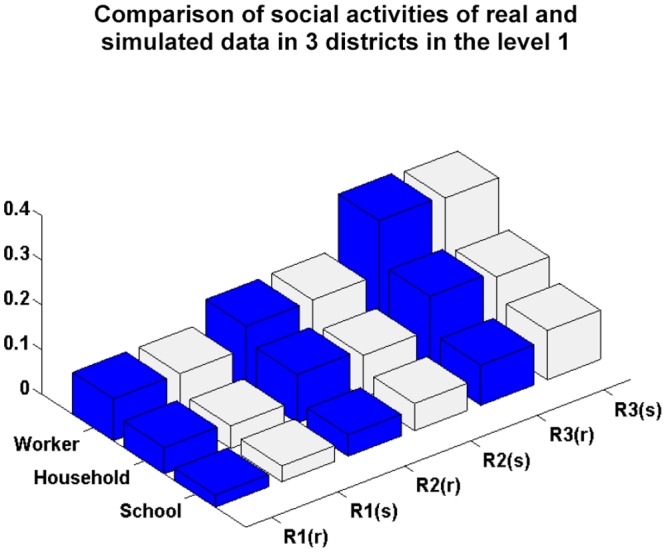
Comparison of the social activities of simulated and real data in the districts at level 1 (where R1(r), R2(r) and R3(r) denote the real data in the three districts, respectively, and R1(s), R2(s) and R3(s) denote the simulated data in the three districts, respectively).

**Fig 6:**
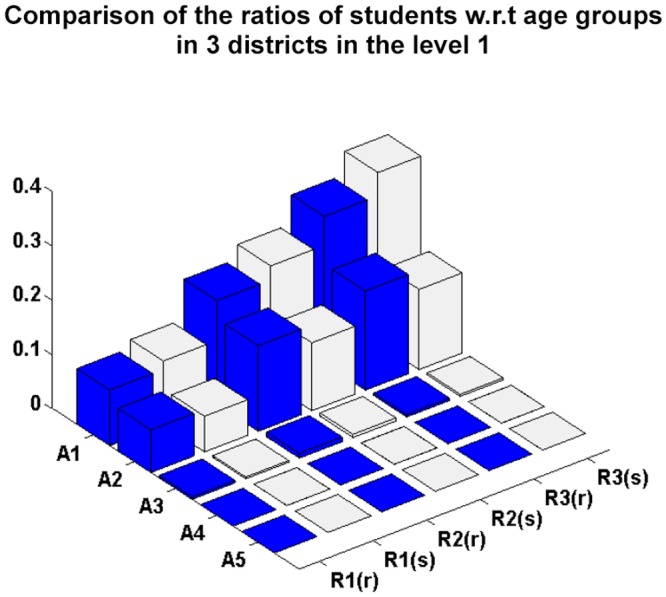
Comparison of the ratios of students in relation to age group for the simulated and real data in the level 1 districts.

**Fig 7:**
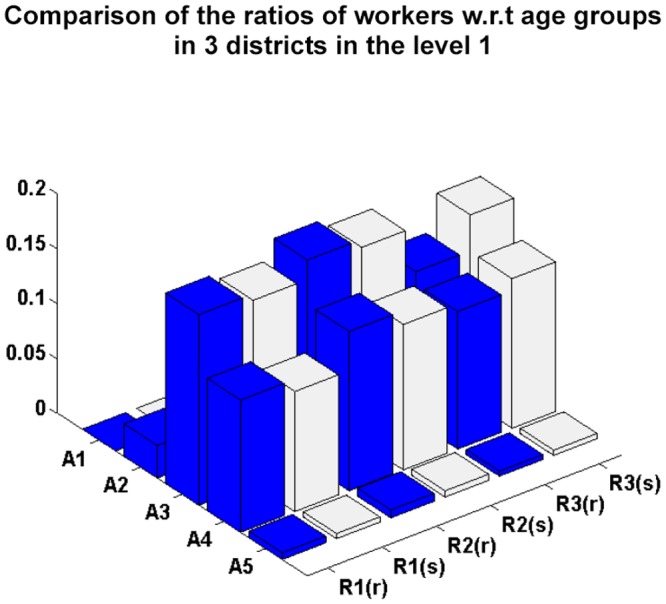
Comparison of the ratios of workers in relation to age group for the simulated and real data in the level 1 districts.

Fig. 5 compares the real and simulated social activity data in the different districts at level 1. In Fig. 5, the number of simulated individuals who stay at home, study or work in correspondence with the number of householders, students and workers, respectively, is close to those of real individuals, indicating that the simulation of individuals social activities satisfies the decision-making requirement. Figs. 6 and 7 compare the ratios of students and workers in relation to the simulated and real age group data in the hierarchical district structure, and show them to be similar. From Figs. 6 and 7, we can conclude that the proposed approach successfully simulates the students and workers in correspondence with the age groups and districts.

We also use a linear regression model with zero intercept between the real and simulated data in Figs. 4, 5, 6 and 7. The values of the coefficient of determination *R* ^2^ are 0.9039, 0.9677, 0.9633 and 0.9781, respectively. This indicates that the difference between the estimated and real data is very small.

### Hierarchical-district-structure-based contact matrices

**Fig 8:**
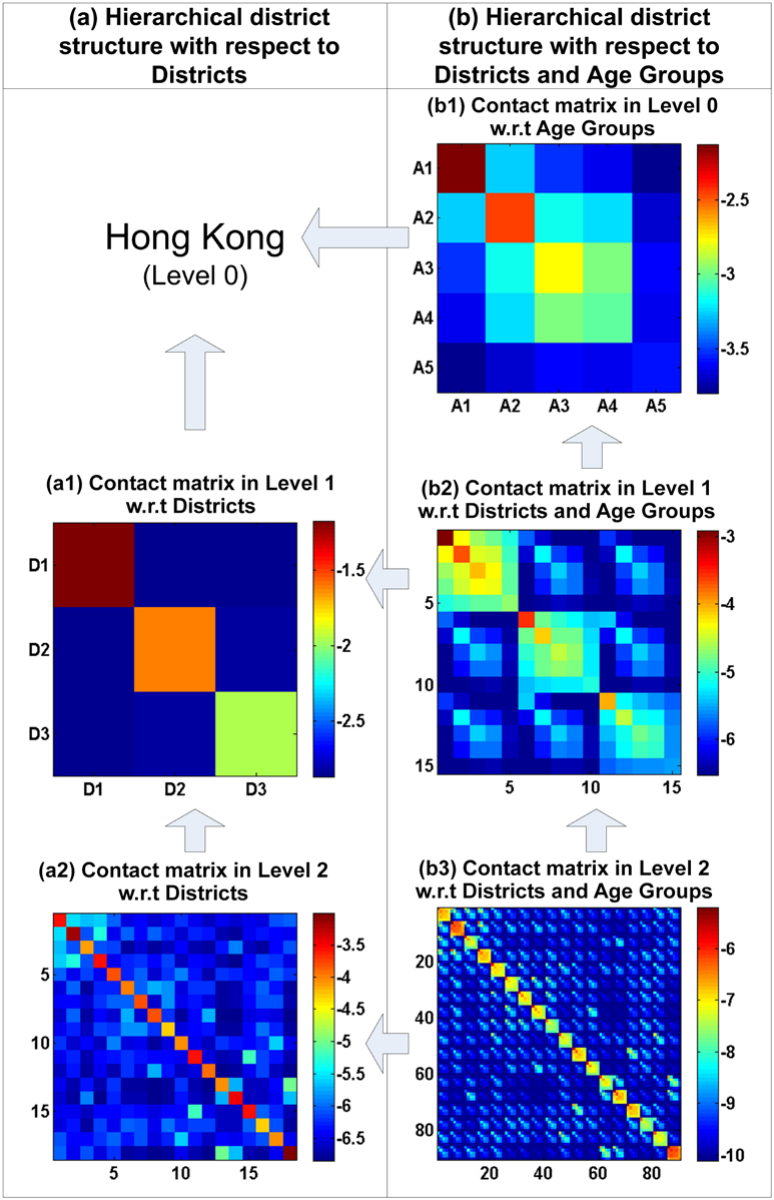
Hierarchical-district-structure-based contact matrices for Hong Kong. (All of the values are shown on a logarithmic scale. (b1) A1, A2, A3, A4 and A5 denote the five age groups (5–14, 15–24, 25–44, 45–64 and 65+, respectively. (b2) The numbers 1, 2, …, 15 denote the combinations of 3 districts and 5 age groups in the level 1, including R1A1, R1A2, …, R3A5, respectively. (b3) The numbers 1, 2, …, 90 denote the combinations of the 18 districts (Central and Western, Wan Chai, Eastern, Southern, Yau Tsim Mong, Sham Shui Po, Kowloon City, Wong Tai Sin, Kwun Tong, Kwai Tsing, Tsuen Wan, Tuen Mun, Yuen Long, North, Tai Po, Sha Tin, Sai Kung, Islands) and 5 age groups in the level 2, including R1A1, R1A2, …, R18A5, respectively.)

Fig. 8 illustrates the hierarchical-district-structure-based contact matrices in Hong Kong, which include district and age-district matrices. Fig. 8 presents the following findings. First, as illustrated in Figs. 8(a1) and (a2), the values of the entries on the diagonal of the district contact matrices are significantly higher than those not on the diagonal. Although the contact matrices in Figs. 8(a1) and (a2) belong to different levels in the hierarchical district structure, they possess the same pattern. This indicates that most of the social contacts appear in the same district, possibly because the social contacts made among individuals in the household and community often occur in the same district. Second, as shown in Fig. 8(b1), the values of the entries in the contact matrices among children and teenagers A1(5–14) and A2(15–24) are higher than those in the contact matrices among other age groups. The same contact patterns can be discovered in Figs. 8(b2) and (b3). Children and teenagers (A1(5–14) and A2(15–24)) in schools have a number of classmates, which may generate many social contacts compared with adults (A3 and A4) and the elderly (A5(65+)). Third, the contact matrix entries that correspond with the same age group in the same district have higher values than those related to different age groups. For example, the contact matrix entries that correspond with the A1, A2 and A3 age groups have the largest values in Fig. 8(b1), and the entries related to the A1, A2 and A3 age groups have larger values than those related to different age groups in the same district, as shown in Figs. 8(b2) and (b3). This means that individuals in social networks prefer to contact individuals within the same age group. For instance, infants make friends in kindergarten, schoolmates prefer to take part in activities with their classmates and workers prefer to be in contact with colleagues. Fourth, as illustrated in Figs. 8(b1) and (b2), the contact probabilities between the individuals in the A4 and A3 age groups are larger than those between the individuals in the A4 group and other groups. The adults in the A4 group have powerful social networks and participate in more social activities with the adults in the A3 group compared with the other age groups. Fifth, as shown in Fig. 8(b1), the values of the contact matrix entries that correspond with the A5 age group are small. Elderly individuals participate in few social activities and only make contact with their sons or daughters in the A3 and A4 groups. In summary, the contact matrices related to age groups and districts at different levels in the hierarchical district structure share similar patterns, reflecting Hong Kongs social and demographic situation.

### Cross-district contact matrices

**Fig 9:**
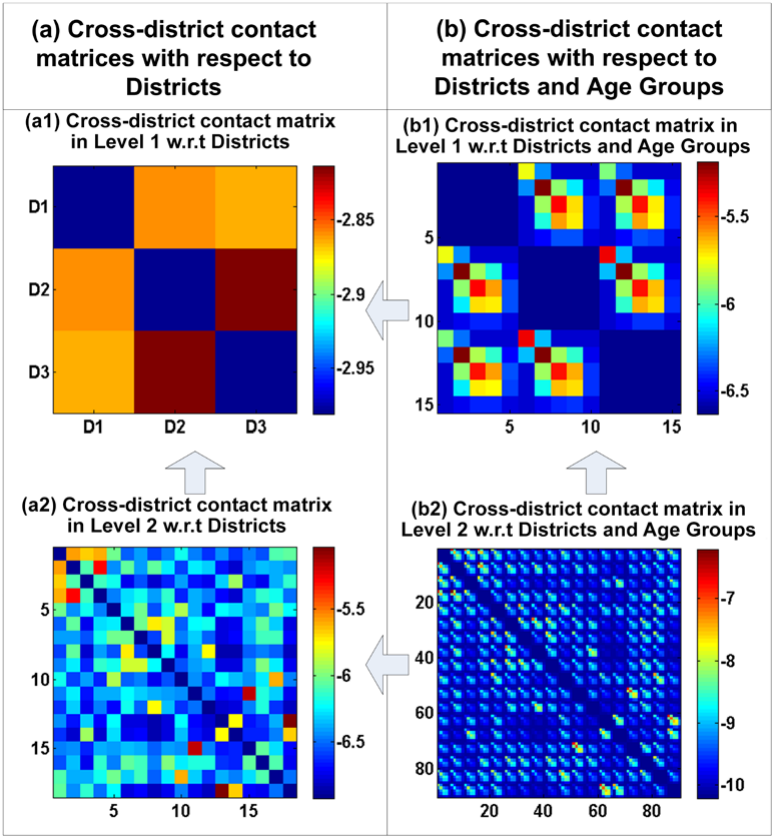
Cross-district contact matrices for Hong Kong. (All of the values are shown in logarithmic scale. (b1) The numbers 1, 2, …, 15 denote the combinations of 3 districts and 5 age groups in the level 1. (b2) The numbers 1, 2, …, 90 denote the combinations of the 18 districts and 5 age groups in the level 2.)

To determine the contact patterns from the cross-district contact matrices, we set the values of all of the entries in the same district to zero. Fig. 9 shows the cross-district contact matrices in Hong Kong, including those that correspond with the district and age-district groups.

Several observations can be made. First, as shown in Fig. 8 (a1), the contact probabilities between the individuals in the neighboring districts are large, such as between the individuals in R2 (Kowloon Peninsula) and R3 (the New Territories) and between the individuals in R1 (Hong Kong Island) and R2. There are several possible reasons for this. (*i*) Most of the individuals who work in the Kowloon Peninsula live in the New Territories, which has a lower cost of living. In addition, the Kowloon Peninsula has more job opportunities and good schools that attract students. (*ii*) Hong Kong Island is a business, cultural and government center. The individuals located there and in the Kowloon Peninsula have more contacts due to the higher prominence of business, cultural and political affairs.

Second, the values of the entries in the upper left corner of the cross-district contact matrix in Fig. 8 (a2) are larger than those in the remaining area of the matrix. The upper left corner of the matrix corresponds with four districts at level 2 of the hierarchical district structure, including the Central and Western, Wan Chai, Eastern, and Southern districts. There are several possible reasons for the high contact probabilities among the individuals in these districts. (*i*) The Central and Western district is Hong Kongs central business and government district. It includes the headquarters of financial services corporations, government headquarters and the consulates of many countries. The Wan Chai district is the heart of Hong Kong. It includes government buildings, art centers, hotels, shopping malls and large exhibition and conference centers. It is reasonable to assume that there is a high probability of contact between the individuals in the Central and Western district and the Wan Chai district. The individuals in the companies or governments establish many social contacts to deal with business procedures between these two districts. (*ii*) The Eastern district has the second highest population among the 18 districts, including many public and large private housing estates. The Southern district has many residential areas. Most of the residents in the Eastern and Southern districts work in the Central and Western district and Wan Chai district, leading to a high probability of contact among these individuals. The most interesting observation is that the contact probabilities between the individuals in the Eastern and Southern districts are lower than those among the individuals in other combinations of the four districts. Both the Eastern and Southern districts are residential areas, encouraging a low contact frequency between the individuals located there.

**Fig 10:**
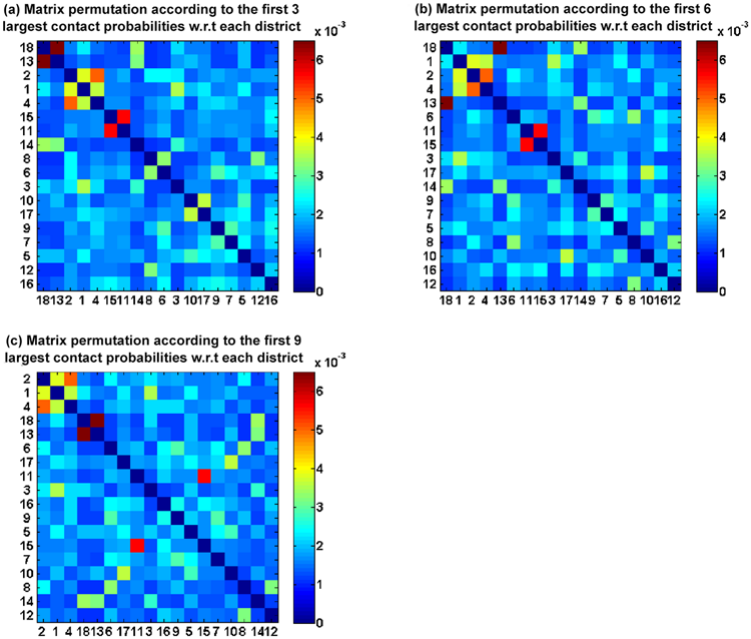
Cross-district contact matrix permutation for each district at level 2. (The numbers 1, 2, …, 18 in (a), (b) and (c) denote the level 2 districts, including Central and Western, Wan Chai, Eastern, Southern, Yau Tsim Mong, Sham Shui Po, Kowloon City, Wong Tai Sin, Kwun Tong, Kwai Tsing, Tsuen Wan, Tuen Mun, Yuen Long, North, Tai Po, Sha Tin, Sai Kung and Hong Kong Island.)

We perform a matrix permutation on the cross-district contact matrix at level 2 in relation to the first *l* largest contact probabilities for each district. Fig. 10 illustrates the cross-district contact matrix permutation at level 2 according to the first 3, 6 and 9 largest contact probabilities for each district, respectively. The Yau Tsim Mong, Sham Shui Po, Kowloon City, Yuen Long and Sha Tin districts, which have index values of 5, 6, 7, 13 and 16, respectively, are always sorted in the first 10 rows as shown in Figs. 10(a), (b) and (c). There are several possible reasons for this. (*i*) The Yau Tsim Mong district is located across Victoria Harbour, which is the business center in the Knowloon City district at level 1. A lot of visitors shop in the Yau Tsim Mong district, which creates a lot of social contacts. (*ii*) The Sham Shui Po district is a commercial, industrial and transportation hub of the territory, and generates a number of social contacts. (*iii*) The Kowloon City district includes four universities, or half of the universities in Hong Kong. Individuals gather together in this district to study, which increases the probability of contact. (*iv*) The Yuen Long district has the youngest population in Hong Kong. The average ages for males and females in this district are 32 and 27, respectively, which means that most of the individuals belong to the A3 age group. (*v*) The Sha Tin district is a living center in Hong Kong, and includes many residents and large residential areas. Most of the residents in the Sha Tin district work in other districts, which leads to a high contact probability.

We further interpret the implications of the cross-district contact matrices for epidemic modeling or control. Assuming an epidemic were to break out in the Central and Western district, the neighboring Wan Chai, Eastern and Southern districts would be affected immediately, as shown in Fig. 10 (a), due to their high contact probabilities with the Central and Western district. The Yau Tsim Mong district would also be affected, as it is closer to and has a higher probability of contact with the Hong Kong Island district compared with other districts. The Sham Shui Po and Kowloon City districts would then be affected in turn. The epidemic would eventually spread to all of the districts in Hong Kong. One way to prevent the spreading of disease in Hong Kong is to provide more vaccinations in the Yau Tsim Mong district, which would be useful in isolating the Hong Kong Island and Kowloon City districts.

In summary, the newly generated cross-district contact matrices not only reflect the situation of the commuter population in Hong Kong and the corresponding social factors, but also reflect the social, economic and cultural situations of the different districts.

### Comparison with a traditional contact matrix for Hong Kong

**Fig 11:**
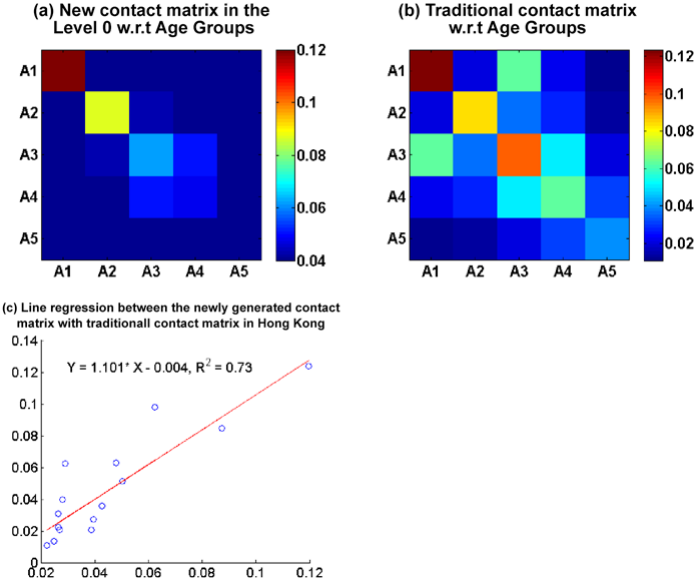
Linear regression between the newly generated and the traditional contact matrices for Hong Kong. (X denotes the values in the traditional contact matrix and Y denotes the values in the newly generated contact matrix.)

The traditional contact matrices for Hong Kong consider only age groups. Hence, we compare the newly generated contact matrix, which corresponds with age groups, with the traditional contact matrix for Hong Kong [31] [32], which is derived according to the approach in [36]. Figs. 11(a) and (b) compare the visualization results of the newly generated and traditional contact matrices, respectively. The two contact matrices share similar patterns. For example, the contact probability in the same age group is higher than those in different age groups in both contact matrices. The order of contact probability in terms of its value in the same age group is also similar between the two matrices. This indicates that the newly generated and traditional contact matrices are similar. We adopt a linear regression model for the traditional and newly generated contact matrices from our model with zero intercept, as illustrated in Fig. 11(c) (where *X* denotes the new contact matrix and *Y* denotes the traditional contact matrix). The coefficient of determination *R* ^2^ is 0.72. The two contact matrices are very similar, but exhibit a small difference that may be caused by the different data-driven models. Our newly generated contact matrix is inferred from the 2011 Hong Kong census data, and the traditional contact matrix is derived from survey data from eight European countries in 2008 [36]. For example, our proposed data-driven model views individuals in the same occupation and industry as a homogeneous unit instead of accounting for the workplace, which lead to a change in contact probabilities among the individuals in the A3 and A4 age groups.

We use the SEIR model to simulate the spread of the infectious virus during the 2009 Hong Kong H1N1 swine flu epidemic. When the first infection case was confirmed by Hong Kongs Center for Health Protection (CHP) on May 2, 2009, the public health department adopted several intervention strategies, such as vaccination, the segregation of infection cases and school closures to control the dynamics of the epidemic. To reasonably validate the proposed epidemic model, we compare the infection case data accumulated between May 23 and July 27, 2009 with the simulation results. The SEIR model adopts the same parameter values adopted in [20] to simulate the dynamics of the H1N1 epidemic in Hong Kong in terms of the size of the accumulated infectious population. We consider five age groups during the simulation, including A1 (5–14), A2 (15–24), A3 (25–44), A4 (45–64) and A5 (65+).

**Fig 12:**
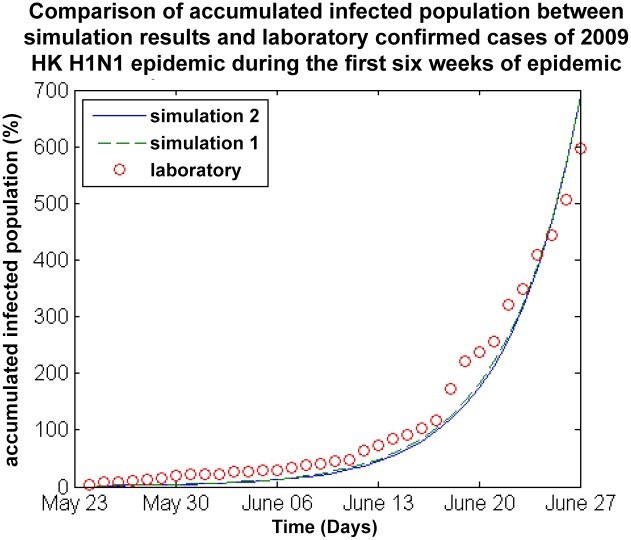
Comparision of the accumulated infected population based on simulation results and laboratory-confirmed infection cases during the first six weeks of the 2009 Hong Kong H1N1 epidemic. (Simulation 1 is based on the newly generated contact matrix and simulation 2 is based on the traditional Hong Kong contact matrix.)

The circle line in Fig. 12 shows the number of infectious cases confirmed in the laboratory by the CHP in practice during the 2009 Hong Kong H1N1 swine flu epidemic from May 23 to July 27, 2009 without any intervention strategies taken. The red dotted line in Fig. 12 illustrates the simulation results obtained by the SEIR model in combination with the new contact matrix. The solid line in Fig. 12 shows the simulation results obtained by the SEIR model with the contact matrix obtained in [20].

Three of the curves in Fig. 12 are close to one another, which means that the simulation results obtained by the SEIR model in combination with the new contact matrix or the contact matrix used in [31] [32] are consistent with the observed dynamics of the infectious virus in practice, especially the dotted line in Fig. 12. The new contact matrix fully reflects the social contacts among the different age groups in Hong Kong, which further improves the simulation results of the SEIR model and gives a more accurate reflection of the spread of the infectious virus. In general, the simulation results of the SEIR model in combination with the new contact matrix qualify for epidemic dynamic simulation, and are suitable for exploring the effect of different vaccine distribution strategies.

**Fig 13:**
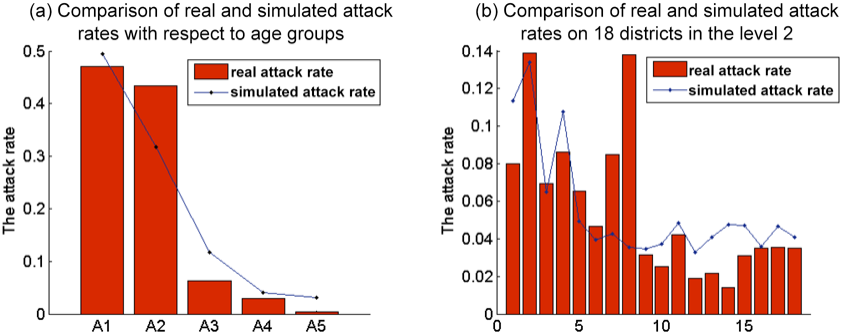
Comparison of the age- and district-specific disease attack rates in Hong Kong.

We also compare the age- and district-specific disease attack rates to explore whether the generated hierarchical contact structure can reproduce the observed dynamics of a spreading disease. The age- and district-specific disease attack rates are defined as follows:

$$\begin{array}{c} \Lambda_{h}=\frac{I_{h}}{\sum_{k}I_{k}} \end{array}$$ (22)

$$\begin{array}{c} \Lambda_{i}=\frac{I_{i}}{\sum_{j}I_{j}} \end{array}$$ (23)

where *h*, *k* ∈ {1, …, *a*}, *a* is the number of age groups, *i*, *j* ∈ 1, …, *r* and *r* is the number of districts. Fig. 13 compares the simulated and real results of the age- and district-specific disease attack rates in Hong Kong. The simulation results are close to the real results, as shown in Figs. 13 (a) and (b), especially the results in the A1 age group in Fig. 13 (a) and the results of the R1 district in Fig. 13 (b). The hierarchical contact structure can improve our understanding of epidemic dynamics.

## Conclusion

In this paper, we investigate the problem of mining social contact patterns from census data. Our major contribution is a newly proposed approach to determining social contact patterns from detailed Hong Kong census data. Compared with previous research, our proposed approach not only captures the hierarchical relationships of social contact patterns in relation to districts at different levels, but also characterizes the social contact patterns among the districts according to age group, allowing social contact patterns to satisfy the requirements of the public health department at different levels. The newly generated contact matrices that use the proposed approach reflect the social contacts within Hong Kongs social, economic and demographic structures and other related factors. We also evaluate the newly generated contact matrix derived from the 2011 Hong Kong census data by conducting simulation-based model experiments and predicting the dynamics of the 2009 Hong Kong H1N1 epidemic. Our experimental results show that the derived contact matrix gives more accurate SEIR-model-based predictions of the spread of disease. In the future, we will include more sociological knowledge in our analyses.
